# Trends in Prevalence and Mortality of Dementia in Elderly Hong Kong Population: Projections, Disease Burden, and Implications for Long-Term Care

**DOI:** 10.1155/2012/406852

**Published:** 2012-10-14

**Authors:** Ruby Yu, Pui Hing Chau, Sarah M. McGhee, Wai Ling Cheung, Kam Che Chan, Sai Hei Cheung, Jean Woo

**Affiliations:** ^1^Department of Medicine and Therapeutics, The Chinese University of Hong Kong, Hong Kong; ^2^Faculty of Social Sciences, The University of Hong Kong, Hong Kong; ^3^School of Public Health, The University of Hong Kong, Hong Kong

## Abstract

*Background*. We describe the trends in prevalence and mortality of dementia among older people in Hong Kong over time. Projections of the number of older people with dementia through 2039 and estimation of the disease burden are also included. *Methods*. Prevalence data were extracted from previous studies in Hong Kong. Mortality data were obtained from the Department of Health of Hong Kong. Projections of the number of people with dementia were calculated by applying the prevalence rates of dementia obtained from previous studies to Hong Kong population projections. The burden of dementia was measured by Disability-Adjusted Life Years (DALYs). *Results*. The number of people aged 60 and above with dementia is projected to increase by 222%, from 103,433 in 2009 to 332,688 in 2039, with a large proportion of those living in institutions. The number of deaths due to dementia among people aged 60 and above has more than doubled between 2001 and 2009. Mortality rates for dementia have also risen. In 2006, about 286,313 DALYS were lost due to dementia. *Conclusions*. The information presented may be used to formulate a long-term care strategy for dementia of the ageing population in Hong Kong.

## 1. Introduction

With population ageing all over the world, the impact of dementia is set to accelerate in the coming years [1], in that it is a chronic disease and is one of the major contributors to disability and increases the burdens to caregivers as well as health and social care systems. In Hong Kong special administrative region (Hong Kong), the population is also ageing rapidly, such that the population aged 60 and above nearly doubled during the past two decades, from 531,600 (10.3%) in 1981 to 1,351,000 (19.2%) in 2011 [2]. It is projected that in 2039, there will be nearly three million people aged 60 and above in Hong Kong [3]. Life expectancy at birth in Hong Kong has also been on the rise for two decades, from 72.3 years for men and 78.5 years for women in 1981 to 80.5 years and 86.7 years in 2011 respectively [4].

It is well known that dementia becomes more prevalent with increasing age; hence, the prevalence of dementia is expected to increase significantly, with substantially increasing disability burden and costs of long-term health and social care. Informal caregivers would need to be an integral part of care since the “oldest old support ratio” (ratio of people aged 50–74 years to people aged 85 years and above) has been decreasing as evidenced in the past two decades due to population ageing [5]. The decreasing ratio implies each informal caregiver is caring for more people aged 85 years and above. Given the expanding population with dementia and the shrinking pool of informal caregivers, a question of how to maintain continual care for such patients arises. Comprehensive long-term care for dementia requires accurate and updated information about trends and burden of the disease. In Hong Kong, a number of studies have investigated the prevalence of dementia [6–8]. However, it remains unclear whether the prevalence of dementia has increased, decreased, or remained stable over time. Furthermore, very few studies examined trends in mortality from dementia in Hong Kong, although it is among the top ten leading causes of death.

In this study we presented trends in prevalence and mortality of dementia among people aged 60 years and above in Hong Kong over a number of years. We projected the number of older people three decades later, as well as estimated the burden of dementia using Disability-Adjusted Life Years (DALYs). We also described the impact of dementia on an ageing population and made recommendations on the health and social care systems for improving long-term care.

## 2. Data

Previous trends in the percentage of older people with dementia were sourced from a series of three household surveys conducted by Census and Statistics Department of Hong Kong in 2000 [9], 2004 [10], and 2008 [11] and from two community studies conducted by Chiu et al. in 1995 [6] and by Lam et al. in 2005/2006 [7, 8]. The latest available estimates of the percentage of older people with dementia, that is, those obtained from the Census and Statistics Department of Hong Kong in 2008 [11] and from the Lam et al. study in 2005/06 [7, 8], were used for the prevalence projection.

The International Classification of Diseases (ICD) code was used for classifying mortality from dementia in Hong Kong. From 2001, deaths due to dementia were identified by the 10th revision of the ICD codes (ICD-10) F01, F03, G30, and G31. Before 2001, deaths due to dementia were identified by the 9th revision of the ICD codes (ICD-9). However, many deaths due to dementia were not coded accordingly in the ICD-9 system [12]. Hence, we studied all deaths due to dementia that occurred in Hong Kong from 2001 and onwards. Annual data on the deaths due to dementia by age and sex between 2001 and 2009 were obtained from the Department of Health of Hong Kong [13]. The mid-year population estimates between 2001 and 2009 and population projections for the Hong Kong population in 2039 were obtained from the Census and Statistics Department of Hong Kong [2, 3]. 

## 3. Method

Age-sex-specific number of people with dementia was estimated for community and institutional populations separately. The total numbers of people with dementia in the community and in institutions in Hong Kong in 2009 and 2039 were estimated by multiplying the age-sex-specific percentage of older people with clinically diagnosed dementia obtained from the Lam et al. study in 2005/06 [7, 8] to the Hong Kong population living in the community; and the percentage of older people with respondent-reported dementia obtained from the Census and Statistics Department of Hong Kong in 2008 [11] to the Hong Kong institutional population. To obtain the population living in the community and institutional population, the proportions of the respective population in Hong Kong were assumed to be constant as those in 2008 [11] and applied to the mid-year population in 2009 and the projected population in 2039 [3]. To assess the trends in mortality, age-adjusted dementia morality rates were calculated for both sexes. Mortality rates were standardised using the direct method to the Hong Kong population as of mid-2009 as the standard [2].

The burden of dementia for people aged 60 or above in the year 2006 was assessed with the DALYs, a summary measure of the burden from premature mortality in terms of years of life lost (YLLs) and the burden from morbidity in terms of years lost due to disability (YLDs). YLLs were calculated based on the age at which the person dies and the life expectancy for people of that age (as determined by a life table) [14]. YLDs from dementia were calculated by multiplying the number of people with dementia in Hong Kong by the disability weight that applies to them. It is assumed that all people with dementia in 2006 experienced their condition for the entire year. Based on the Dutch weights from the Global Burden of Disease study of WHO [15], disability weights for mild, moderate, and severe dementia were 0.27, 0.63, and 0.94, respectively. Because disability weights for dementia were defined for different levels of severity of dementia, we calculated the average disability weight for the estimation of YLDs. Based on a local study conducted by Chiu et al. [6] the proportions of mild, moderate, and severe dementia were 65.6%, 25.0%, and 9.4%, respectively. Therefore, combined with the disability weights listed above, the average disability weight for dementia was calculated by multiplying the proportion of mild, moderate, and severe dementia with the corresponding disability weight, hence giving an average of 0.42. The YLDs due to dementia was thus calculated by multiplying the number of people with dementia by the disability weight and the life span with dementia.

## 4. Results

### 4.1. Trends in Prevalence Rates of Dementia

According to respondent-reported data, the percentage of community-dwelling people aged 60 and above in Hong Kong with dementia increased from 0.6% in 2000 [9] to 1.1% in 2004 [10], and remained stable until 2008 (1.1%) [11]. The percentage of community-dwelling people aged 70 and above with clinically diagnosed dementia also increased from 4.5% in 1995 [6] to 9.3% in 2005-2006 [7, 8]. The percentage of people with clinically diagnosed dementia increased with age and approximately doubled for every five years until around age 90. It was also observed that a higher percentage of females have dementia than males at older ages.

The percentage of people living in institutions with dementia was higher than that people living in the community, with 30.7% of institutionalized people aged 60 and above reported (by proxy respondents) having dementia in 2004 [10] and 2008 [11] and 17.4% aged 70 and above were found to have clinically diagnosed dementia in 1995 [6]. Owing to the limited data, it was unable to test for the trends statistically. However, the estimates suggested a probable increasing trend in prevalence.

### 4.2. Estimated and Projected Prevalence of Dementia

**Figure 1:**
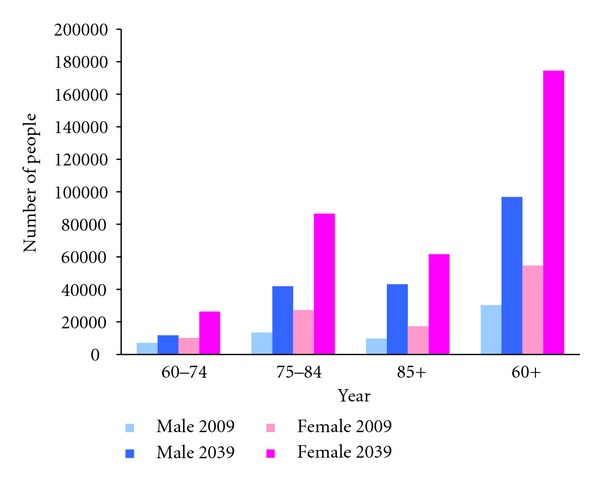
Estimated and projected numbers of people living in community with dementia in Hong Kong, by age group and sex, 2009 and 2039. Data sources: authors' calculations based on data from elderly commission of Hong Kong [7] Lam et al. [8], and Census and Statistics Department of Hong Kong [3].

**Figure 2:**
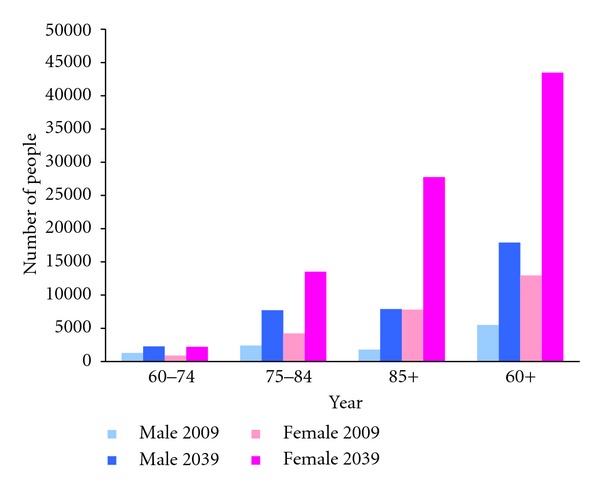
Estimated and projected numbers of people living in institutions with dementia in Hong Kong, by age group and sex, 2009 and 2039. Data sources: authors' calculations based on data from Census and Statistics Department of Hong Kong [3, 11].

Having applied the age-sex-specific percentage of older people with dementia to the Hong Kong population, an estimated 85,012 people aged 60 and above living in the community had dementia in 2009 of whom over 30% were aged 85 and above. This number is projected to increase to 271,320 people in 2039 (Figure 1). For the institutional population, an estimated 18,421 people aged 60 and above were living with dementia in 2009. By 2039, this is projected at 61,367 people (Figure 2). Combining the community and institutional populations, the estimated number of people aged 60 and above with dementia would increase from 103,433 in 2009 to 332,688 in 2039, an increase of 222%.

### 4.3. Trends in Mortality Rates of Dementia

**Figure 3:**
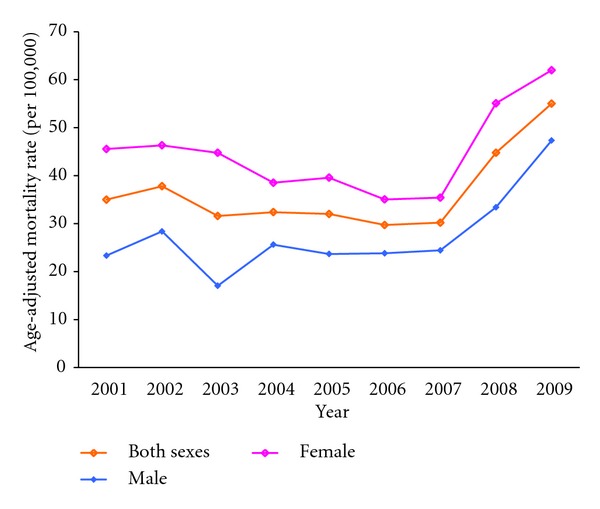
Age-adjusted* mortality rates for dementia (per 100,000) among people aged 60 and above in Hong Kong, by sex, 2001–2009. *The age-adjusted mortality rates used the Hong Kong population as of mid 2009 as the standard. Data sources: authors' calculations based on data from Department of Health of Hong Kong [13].

Deaths due to dementia among the people aged 60 and above had more than doubled over the past 9 years, accounting for 2% of all deaths in 2009 compared to 1% of all deaths in 2001. The age-standardized mortality rate among people aged 60 and above remained stable between 2001 and 2007, but increased sharply in 2008 and 2009 (Figure 3). The rates between 2001 and 2009 increased by 103% for males (from 23.3 per 100,000 to 47.3 per 100,000) and 36% for females (from 45.6 per 100,000 to 62.0 per 100,000). The mortality rates increased exponentially with age. In 2009, while males aged 60 to 84 had a higher age-specific mortality rate from dementia than their female counterparts, the reverse was observed for those aged 85 and above.

### 4.4. Estimated DALYs Lost due to Dementia

The DALYs lost due to dementia for people aged 60 or above in 2006 was 286,313. The majority of burden was due to disability, with YLDs making up 99% of DALYs. The remaining 1% of the burden was due to the YLLs from dementia. The burden of disease from dementia is disproportionately carried by women. While the burden for males was 83,051 DALYs (29% of total), the female burden was 203,262 DALYs (71% in total). Males experienced 839 YLLs and 82,212 YLDs, while females experienced 1,148 YLLs and 202,114 YLDs. 

## 5. Discussion

Several population-based observational studies have reported secular trends in the prevalence of dementia worldwide. However, trends in dementia prevalence vary markedly from one study to another [16–20], and there have been few data on the trends in prevalence of dementia in Hong Kong. Based on existing local studies, an increasing trend in the percentage of people with dementia was observed in Hong Kong. We also projected that within 30 years, the number of people aged 60 and above with dementia will more than triple, from 103,433 people in 2009 to 332,688 people in 2039. Recent data from the Rotterdam Study revealed that the incidence of dementia is declining, perhaps because of preventive measures and better control and treatment of vascular risk factors [21] and therefore the prevalence of dementia may increase more slowly than expected. However, an increasing trend was observed for the prevalence of hypertension in Hong Kong over the past years, especially in the middle-aged group [22, 23] which may probably lead to an increasing trend in prevalence of vascular dementia in the future. The increasing burden indicates the challenges ahead and more should be done to improve the long-term care for people with dementia and their caregivers.

The increasing prevalence of dementia suggests the need for urgent plans for better long-term institutional care system with dementia-specific services. In Hong Kong, most of the institutions are not geared to meeting the distinct needs of people with dementia and therefore access of people with dementia and their caregivers to adequate services is limited. Quality of care, autonomy, and dignity of institutional care are also important issues that need to be addressed. Periodic reports in the media in the past have emphasized undesirable behavior leading to abuse. The quality of care depends on a large extent on adequate staff numbers [24]. However, there is not sufficient number of staff to meet many of the residents' needs in Hong Kong and it is recognized that there is a tension between quality and affordability, which is unlikely to be resolved until the financing of long-term care is addressed. There is also a shortage of well-trained staff. Heath care professionals in institutional care were generally inadequately trained in dementia needs. Up to standard training must be developed for all these people to cater for a more effective and high-quality care for people with dementia. The lack of staff continuity is another issue that needs to be resolved. With the increasing prevalence of dementia, more well-trained professionals would be required. Career development, promotion opportunity, training, and recognition should be provided to encourage more new professionals. 

There also needs to be an improvement in the end-of-life care for people with dementia. However, palliative and hospice care services are not particularly well developed in Hong Kong, with few comprehensive services provided in hospital settings. A survey in a nonacute hospital in Hong Kong revealed that those with dementia tend to have more nonpalliative interventions compared to patients with cancer [25] as had been noted in a previous study of nursing home residents in the United States [26]. It has been pointed out that palliative and hospice care could greatly improve the care of patients with advanced dementia resulting in better quality of life, greater caregiver satisfaction, and at the same time, reducing hospitalisation [27]. Therefore, there is a need to raise awareness to improve the quality of care for such patients. Recently, a continuous quality improvement initiative aimed at improving the quality of end-of-life care for noncancer patients had been developed and integrated into part of the care plain in a nonacute hospital in Hong Kong [28]. Evaluation of its impact on patients and their caregivers is warranted.

Informal caregivers will also remain a core part of the long-term care for dementia. However, being an informal caregiver can be detrimental to both the physical and psychological health as well as health-related quality of life [29, 30]. Therefore, both practical and psychological support for caregivers of dementia patients would need to be improved. Support and counselling services should be provided from the moment of diagnosis as the financial and emotional impact on people with dementia and their families can be enormous. It has been recognized that enhanced counselling and support treatment may reduce caregivers' depression [31]. Collaborative care management including education on communication and coping skills, legal and financial advice, patient exercise guidelines, and caregiver guide has also been shown to improve the quality of care and behavioural and psychological symptoms of dementia for both people with dementia and their caregivers [32].

Standardized to the World Health Organization population, the age-adjusted mortality rate for dementia of all ages in Hong Kong in 2008 (3.5 per 100,000 population) was lower than the corresponding rates in the United States (24.8 per 100,000 population), the United Kingdom (17.1 per 100,000 population), and Australia (15.3 per 100,000 population), comparable to China (3.2 per 100,000 population), but higher than Japan (2.5 per 100,000 population) and Singapore (0.02 per 100,000 population) [33]. The increasing trend in age-adjusted dementia mortality rates in Hong Kong since 2007 may be attributed to several factors, including increasing awareness, increased diagnosis, and population ageing. Sharp increase has also been observed in Australia since 2006 [34] where some deaths that were previously coded as cerebrovascular disease were coded as vascular dementia due to change in coding instructions. This change may be explained by the increased awareness of dementia. Perhaps this phenomenon occurred in Hong Kong with some time lag.

The increased mortality rates highlight the importance of early detection and cognitive assessment of the disease. Currently diagnosis tends to be made at a later stage of the disease, with up to 90% of people with mild dementia never receiving a diagnosis. To encourage early detection, competencies of early diagnosis of dementia should be developed and cognitive function should be a regular feature of health assessment for the elderly in primary care. The findings of this study also suggest that dementia is a leading cause of disability in older people in Hong Kong and the burden associated with it is substantial, as with China and other countries [35]. Nevertheless, limited awareness of dementia and/or denial of its existence, and stigma attached to the condition, may lead to excess disability. Therefore public education and training of health-care professionals and caregivers are needed in raising awareness of the disease and its management.

There are limitations in this study. The data in this study were compiled from different sources, some studies reporting rates for Alzheimer's disease while others reported other forms of dementia. Therefore we have presented the trends for all these categories combined. Trends for the subtypes of dementia could not be investigated. There is a possibility of diagnosis being affected by change in awareness of the disease, such that increasing awareness may partly account for the increasing trend in prevalence. Like other self-reported data, respondent-reported data are subject to reporting error. Nevertheless, it is a commonly used method in household surveys. As for the projections, we used constant age-sex-specific prevalence rates of clinically diagnosed dementia for the projections which reflect the impact of demographic ageing alone. However, percentage of people with dementia appears to have increased and therefore the number of people with dementia is expected to increase even faster. There may be some underreporting of dementia as a cause of death, with many deaths incorrectly attributed to pneumonia or other causes. To tackle this, the attributable risk methodology could be adopted. However, the relative risk of death established by Mathers and Leonardi in 2003 [36] may not apply to the recent deaths with the sharp increase of mortality coded as dementia since 2008. Therefore, mortality statistics need to be interpreted with caution. Finally, we did not have information regarding factors that contributed to trends in the prevalence and mortality of dementia. Efforts need to be made to monitor the secular trends of various predisposing risk factors.

In conclusion, dementia is a significant health and social problem in Hong Kong. The increasing trends in prevalence and mortality of dementia and its high-disability burden have an enormous impact on the health care and social services systems. Therefore the formulation of a dementia care strategy as part of a long-term care strategy for the elderly would be important for Hong Kong.
